# Job demands and general health of nursing staff in German nursing homes during the COVID-19 pandemic: the buffering effect of job resources

**DOI:** 10.1186/s12912-025-03924-x

**Published:** 2025-10-06

**Authors:** Anna Hirschmüller, Aline Wege, Pavel Dietz, Albert Nienhaus, Elisabeth Diehl

**Affiliations:** 1 Institute of Occupational, Social and Environmental Medicine, University Medical Center of the Johannes Gutenberg University Mainz, Obere Zahlbacher Str. 67, 55131 Mainz, Germany; 2 Department for Occupational Medicine, Hazardous Substances and Health Science, Institution for Accident Insurance and Prevention in the Health and Welfare Services (BGW), Pappelallee 33/35/37, 22089 Hamburg, Germany

**Keywords:** Job demands, Resources, Occupational health, Nursing staff

## Abstract

**Background:**

Nursing staff in nursing homes face high workloads, negatively impacting their health. This issue is exacerbated by workforce shortages and rising care needs, highlighting the necessity of identifying resources that mitigate workload-related stress.

**Methods:**

A cross-sectional survey was conducted among 364 nurses from 55 nursing homes in Rhineland-Palatinate, Germany, at the end of 2021 during the COVID-19 pandemic. Quantitative demands, personal and job-related resources and general health were assessed using the Copenhagen Psychosocial Questionnaire (COPSOQ), the Brief Resilience Scale (BRS), and the German general self-efficacy short scale (ASKU). Moderator analyses were conducted to examine the buffering effect of resources on the impact of job demands on general health.

**Results:**

High quantitative demands were significantly associated with poorer general health among nursing staff. Job-related resources, including sense of community (β = 0.27, *p* < .001), support at work (β = 0.12, *p* = .031), and commitment to workplace (β = 0.13, *p* = .013), moderated the negative effect of job demands on general health. Personal resources such as resilience and self-efficacy did not emerge as significant moderators.

**Conclusions:**

Results show that nursing staff with higher levels of job resources experienced less deterioration in general health under increasing job demands. This demonstrates the importance of fostering organizational resources to mitigate the adverse health effects of high workload in this context. Therefore, systemic organizational changes may be more impactful than personal resource-focused strategies in addressing stress for nursing staff. Interventions that enhance workplace cohesion, support, and commitment are likely to yield benefits for nurses’ health.

## Background

The relevance of nursing staff in nursing homes has become increasingly prominent in recent years, particularly in light of the ongoing workforce shortages that threaten the quality of care provided to the elderly population. As demographic change leads to a growing number of older adults requiring assistance, the role of nurses is critical for ensuring adequate support and maintaining the well-being of residents. With reference to 2021, there is an expected increase by 37% of people in need of long-term care in Germany by 2055, primarily due to the aging population [1]. In addition to demographic changes, Germany faces a shortage of nursing staff caused by unattractive working conditions, below-average pay and lack of appreciation [2]. A nursing workforce projection shows that Germany is expected to face a shortage of at least 280,000 nursing staff by 2049 [3]. These developments are not limited to Germany, but are a global problem [4] and emphasize the importance of efficient nursing staff. However, nursing staff often faces high levels of occupational stress stemming from various factors that involve a high physical as well as mental workload. Previous research indicates that nursing staff is exposed to high workload, emotional demands, physical demands, and time pressure [5–7]. A study by Otto and colleagues [8] identified time pressure as the most straining factor for nurses. This shows that there is a discrepancy between the tasks at work that need to be completed and the time available for it. Compared to nursing staff in elderly care, hospitals and trainees, nurses in home care showed the highest levels of stress [8]. High demands derive from the complex care needs of residents due to multi-morbidity, physical demands due to heavy lifting, and a high nurse-patient ratio due to staff shortages [9–11]. The COVID-19 pandemic intensified existing stressors, with nurses facing heightened physical and emotional demands [12]. During the first wave of the COVID-19 pandemic in Germany, almost 95% of the nurses reported an increase in work demands since the pandemic [13]. Among other things, time pressure, high workloads, and emotional demands have been exacerbated during the pandemic [14, 15].

Research on occupational stress often distinguishes between stressors and strain, where stressors refer to external job demands, such as workload or time pressure, and strain represents the individual psychological or physiological response [16]. As research on the stressor-strain relationship reveals, workload is positively associated with strain [17]. Stressors at work can therefore negatively affect nurses’ health and well-being. Results of meta-analyses demonstrate that occupational stressors are related to physical and psychological symptoms [18, 19]. The adverse effects of workload are more prominent for care workers than for other professionals [17]. Among nurses, stress has been linked to a variety of health problems, including musculoskeletal disorders, anxiety, depression, and burnout [6, 7, 20]. For nursing staff in long-term care facilities the strain is particularly high. For example, Korbus and colleagues [5] showed that 75% of nurses in nursing homes are chronically stressed. The impact of the high strain of nurses is reflected in absenteeism data. In 2023, the absenteeism of nursing staff in Germany was at a record high (28 days) [21]. Geriatric nursing staff had an even higher rate of sick leave (34.2 days) and the rate was 84% higher than for all employed individuals in Germany (18.6 days). This data emphasizes the need for health promotion and improved working conditions [5].

Moreover, the stressors faced by nursing staff, such as time pressure, contribute to emotional exhaustion, which can impair their ability to provide high-quality care [14]. This high stress situation of nurses is compounded by the fact that stressors not only diminish health but also increase the likelihood of staff leaving the profession altogether, further exacerbating workforce shortages in this critical area [22]. So, the implications of high occupational stress extend beyond the health of nursing staff to impact patient care quality and organizational effectiveness.

To mitigate the effects of high workload on nurses’ health, it is essential to explore various personal, social, and organizational resources that can buffer against occupational stress. Previous studies showed that an effective management of job resources – such as supportive leadership, fair management practices, and positive interpersonal relationships – can significantly alleviate stress [6]. Studies show that effective coping strategies are essential; however, only 43% of nurses exhibit health-friendly coping patterns [5]. Social support from colleagues and supervisors and a strong sense of community within the team have been repeatedly identified as key organizational resources that reduce burnout and improve nurses’ health and job satisfaction [6, 7, 23, 24]. Recognition and workplace commitment have been shown to mitigate stress and enhance job satisfaction [6, 23, 24], while a strong meaning of work contributes to lower levels of emotional exhaustion [6, 24, 25]. In addition to these job-related resources, personal resources such as self-efficacy and resilience have been associated with health among nurses [5, 26, 27]. Taken together, these findings provide empirical support for focusing on these specific job-related and personal resources as potential moderators of the impact of quantitative job demands on general health in this study.

The Job Demands-Resources (JD-R) model provides a valuable theoretical framework for understanding the interplay between job demands and resources and their impact on various work and health related outcomes. According to this model, high job demands such as a high workload can lead to strain and health impairment, while adequate resources can promote motivation and well-being [28]. When job demands are high and resources are insufficient to cope with these demands, employees are likely to experience stress, exhaustion, and a decrease in their health.

The aim of the present study was to examine the buffering effect of job-related and personal resources on the impact of job demands on general health of nurses in nursing homes who are, as presented, a professional group experiencing a high workload. Specifically, we hypothesize that higher job demands are negatively associated with general health. Since previous studies showed that a high workload and time pressure are among the most straining factors for nurses [5, 8], stressors were operationalized as quantitative demands in this study. Additionally, we expect that resources will moderate this relationship by buffering the negative effects of high job demands on general health. Personal as well as job resources have been identified as helpful in alleviating stress [23–26]; therefore, both areas of resources were investigated in this study. This focus on internal as well as external elements offers insights into both prevention strategies that focus on individual and those that address structural factors.

While there is a growing body of literature examining the role of resources in reducing stress, there is still a lack of comprehensive studies focusing specifically on the context of nursing homes. The current literature has predominantly focused on emotional exhaustion and burnout as the primary outcomes of high job demands. The objective of this study was to broaden this perspective by examining general health as an outcome and various resources among nursing staff in nursing homes. By specifically focusing on this context, the study aimed to provide insights into how internal and external resources can support nursing staff in managing their workloads effectively, thereby promoting their overall health and well-being. The results will contribute to the development of interventions aimed at improving working conditions for nursing staff in long-term care settings, thereby benefiting both caregivers and the residents under their care.

## Method

### Study design and sample

The reporting of the study adheres to the STROBE checklist [29], which was systematically applied to ensure completeness and transparency of the methodology and results. After receiving approval by the Ethics Committee of the State Chamber of Medicine in Rhineland-Palatinate (clearance number 2020–15537), a cross-sectional observational study was conducted in Rhineland-Palatinate from September to December 2021. The data collection took place during the fourth wave of the COVID-19 pandemic in Germany with the highest 7-day incidence so far at this time [30].

To ensure sufficient power for the statistical analyses, an a prior sample size calculation was conducted. Approximately 26,000 nurses were employed in nursing home in Rhineland-Palatinate [31]. Based on a 95% confidence level and a 5% margin of error, the minimum required sample size is 379. Taking into account an expected response rate of 20%, it would be necessary to distribute at least 1,895 questionnaires. Through an extensive internet search, 506 facilities in Rhineland-Palatinate were identified. A random sample of 25% was drawn. The 55 selected facilities were subsequently contacted via email and telephone. The facility or nursing service managers who agreed to participate in the study provided information regarding the number of nursing staff employed. The corresponding amount of paper-and-pencil questionnaires was distributed to the facilities using postage-paid return envelopes, allowing participants a period of three weeks to complete and return the questionnaires at their convenience. To enhance response rates, a reminder email was sent prior to the deadline. Participation in the study was voluntary and anonymous, with written informed consent obtained at the beginning of the questionnaire. The most frequent cited reason of the facility or nursing service managers for non-participation was reported as nurses having a too high workload to engage in the study.

### Measures

The questionnaire was developed based on findings from a qualitative pre-study [15]. It originally included both self-developed work-related items and validated instruments such as the German standard version of the Copenhagen Psychosocial Questionnaire (COPSOQ) Version III [32], the German general self-efficacy-scale [33], and the German version of the Brief Resilience Scale [34]. Only the validated instruments were used for analyses presented in this study and all instruments were validated German-language versions.

**Job Demands.** Demands at work were measured using the five-item scale *quantitative demands* of the COPSOQ. Prior research has shown that the COPSOQ has adequate reliability as well as content and construct validity [35]. The participants were asked about requirements of their job. An example item includes ‘Do you have to work very fast?’. Response options ranged from 1 = always to 5 = never/hardly ever. The COSPOQ scales utilized in this study were prepared in accordance with the established COPSOQ guidelines [32]. The scale score is calculated as the mean of the items within each scale and then transformed to a 0-100 scale. A score is considered missing if less than half of the items are answered. High values in *quantitative demands* indicate greater demands and are viewed negatively.

**Job Resources.** To measure resources at work the COPSOQ scales *sense of community* (2 items, e.g.: ‘Is there a good atmosphere between you and your colleagues?’), *support at work* (4 items, e.g.: ’How often do you get help and support from your colleagues, if needed?’), *commitment to workplace* (2 items, e.g.: ‘Are you proud of being part of this organization?’), *meaning of work* (2 items, e.g.: ‘Is your work meaningful?’), and *recognition* (1 item: ‘Is your work recognized and appreciated by the management?’) were used in this study. Response options ranged from 1 = always to 5 = never/hardly ever for the scales *sense of community* and *support at work* and from 1 = to a very large extent to 5 = to a very small extent for the scales *recognition*, *commitment to workplace*, and *meaning of work*. The job resources scales were prepared analogously to the scale *quantitative demands*. The scale scores were calculated as the mean of the items within each scale and then transformed to 0-100 scales. A score is considered missing if less than half of the items are answered. High values in the scales measuring *sense of community*, *support at work*, *commitment to workplace*, *meaning of work*, and *recognition* are interpreted positively.

**Personal Resources.** In addition, the German general self-efficacy short scale (ASKU) [33] and the German version of the Brief resilience scale (BRS) [34] were used in this study to operationalise personal resources. The BRS is a reliable and valid instrument to assess resilience [36]. The scale comprises six items to measure the ability to recover from stress (e.g. ‘I tend to bounce back quickly after hard times.’). The response format corresponds to a five-point scale from 1 = strongly disagree to 5 = strongly agree. Three of the six BRS items, that are worded negatively, were reversed and then by averaging all items a total score between 1 and 5 was calculated. A higher score indicates a higher level of resilience. The ASKU is a reliable and valid tool for assessing self-efficacy [33]. Three items are comprised in the scale to measure individual competence expectations addressing challenges with a five-point response format from 1 = strongly disagree to 5 = strongly agree (e.g. ‘I can rely on my skills in difficult situations.’). The mean scale score of the ASKU was calculated by averaging all items resulting in a total score between 1 and 5. A higher score indicates a higher level of self-efficacy.

**General Health.** To assess general health, the COPSOQ scale *general health* was employed. Prior research has demonstrated high sensitivity and validity of this single-item measure [32, 35] as well as nomological validity, showing relevant associations with job demands, resources, and well-being outcomes, consistent with its use as an outcome in the JD-R model [37]. Participants were asked how many points they give their present state of health if they evaluate the best conceivable state of health at 10 points and the worst at 0 points. A high score is viewed positively.

**Control variables.** According to Spector and Brannick’s [38] discussion on the use of statistical control variables, variables potentially related to study measures were controlled for. Age, gender, professional experience, professional qualification, and leadership role were identified as variables that are related to stress [39] and health [40, 41] of nurses in past research, and were therefore included as control variables for all analyses in this study.

### Data analysis

Descriptive statistics were computed to characterise all study variables. To examine associations between all study variables, Pearson correlations were calculated for continuous-continuous variable pairs, point-biserial correlations were used for continuous-dichotomous pairs, and chi-square tests were applied for dichotomous-dichotomous pairs. Pearson correlations were conducted to identify resources associated with general health in bivariate analyses. All resources that showed a significant correlation with general health were subsequently analysed as moderators. Statistical analyses were conducted using SPSS version 29.0.

For moderator analyses, the PROCESS macro developed by Hayes version 4.2 was employed. Moderation analyses were conducted separately for each resource to examine its specific buffering effect on the association between quantitative demands and general health. This approach was chosen to facilitate interpretability and practical applicability of the buffering effects and reduce potential issues with multicollinearity among moderators. To enhance interpretational clarity, all scales were mean-centered as recommended by Hayes [42]. Missing data were handled using listwise deletion. Bootstrapping as a robust method was applied with 5,000 samples as well as HC3 standard errors to report 95% confidence intervals [43]. The linearity of relationships among all variables involved in the moderation analysis was evaluated through visual inspection of scatterplots following LOESS smoothing. Covariates included age, gender, professional experience, professional qualification, and leadership role.

## Results

### Descriptive statistics

Of the 2,448 questionnaires sent out, 404 were returned resulting in a response rate of 16.5%. The final sample consisted of 364 participants after data cleaning. The primary reason for exclusion was missing data on the outcome variable general health. Table 1 presents socio-demographic data of the sample. The participants had an average age of 42.6 years, the majority was female (80.5%). In terms of professional qualifications, 53.3% of the participants were nurses (including participants with university diploma) and 16.5% were nursing assistants. Regarding professional experience, 70.6% of the sample had five or more years of experience. The majority (73.9%) did not have a leadership role.

**Table 1:** Demographic and work characteristics of study participants (*n* = 364)

Characteristic			
Age (mean, SD, range)		42.6, 12.7, 18–76	
Gender (n, %)	Female	293	80.5
	Male	64	17.6
Professional qualification	Nurse	194	53.3
(n, %)	Nursing assistant	60	16.5
	In training	17	4.7
	Other qualification	40	11.0
	No qualification	35	9.6
Professional experience	≥ 5 years	257	70.6
(n, %)	< 5 years	73	20.1
Leadership role (n, %)	Yes	84	23.1
	No	269	73.9

Note. “Other qualification” includes non-nursing staff working in care (e.g. care assistants, everyday companions). The key criterion was employment in nursing care, regardless of formal qualification

In Table 2, the means (*M*), standard deviations (*SD*), and correlations of all variables of this study are presented. Except for the 1-item scales *general health* and *recognition*, Cronbach’s α is reported on the diagonal. Correlation analyses reveal significant associations among the variables. Quantitative demands are negatively associated with resources such as support at work (*r* = − .26, *p* < .01) or commitment to workplace (*r* = − .26, *p* < .01) and with general health (*r* = − .35, *p* < .01). In turn, general health is positively associated with resources such as commitment to workplace (*r* = .27, *p* < .01), sense of community (*r* = .27, *p* < .01), recognition (*r* = .26, *p* < .01), and support at work (*r* = .25, *p* < .01).

**Table 2:** Descriptive statistics and correlations for study variables

Variable	M	SD	1	2	3	4	5	6	7	8	9	10	11	12	13	14
1. Age	42.6	12.7														
2. Gender	0.82	0.38	0.18**													
3. Professional qualification	2.02	1.41	0.04	0.04												
4. Professional experience	0.78	0.42	0.30**	.21^1^	− 0.26**	-										
5. Leadership role	0.24	0.43	0.02	.09^1^	− 0.32**	0.40**^1^	-									
6. Quantitative demands	66.0	20.6	− 0.05	0.01	− 0.14**	0.06	0.08	(0.86)								
7. Sense of community	75.2	19.9	0.01	0.16**	− 0.02	− 0.04	− 0.001	− 0.15**	(0.86)							
8. Support at work	67.7	23.4	− 0.05	0.09	− 0.05	− 0.09	0.06	− 0.26**	0.56**	(0.86)						
9. Commitment to workplace	66.9	25.6	− 0.17**	0.02	− 0.11*	− 0.03	− 0.08	− 0.26**	0.36**	0.46**	(0.76)					
10. Meaning of work	89.4	16.4	− 0.10	0.05	− 0.09	− 0.05	− 0.17**	− 0.16**	0.31**	0.37**	0.45**	(0.82)				
11. Recognition	50.5	29.3	− 0.05	− 0.04	− 0.003	− 0.03	− 0.03	− 0.20**	0.30**	0.50**	0.50**	0.19**	-			
12. Resilience	3.09	0.63	0.02	− 0.001	− 0.14**	0.07	0.20**	− 0.18**	0.12*	0.22**	0.16**	0.08	0.15**	(0.65)		
13. Self-efficacy	3.97	0.68	0.16**	0.04	− 0.09	0.20**	0.07	− 0.17**	0.15**	0.10	0.08	0.19**	0.03	0.22**	(0.87)	
14. General health	54.8	22.6	− 0.09	0.01	− 0.002	− 0.08	− 0.003	− 0.35**	0.27**	0.25**	0.27**	0.20**	0.26**	0.22**	0.15**	-

Note. Gender (0 = Male; 1 = Female). Professional qualification (1 = Nurse; 2 = Nursing assistant; 3 = In training; 4 = Other qualification; 5 = No qualification). Professional experience (0 = less than 5 years; 1 = at least 5 years). Leadership role (0 = No; 1 = Yes). Cronbach’s α in parentheses.^1^φ-coefficients are reported for dichotomous variables. *p < .05. **p < .01

### Moderator analyses

Moderator analyses were conducted to examine the buffering effect of job and personal resources on the impact of job demands on general health. Quantitative demands were included as independent variable, general health as dependent variable and sense of community, support at work, commitment to workplace, meaning of work, recognition, resilience, and self-efficacy as moderators. Age, gender, professional qualification, professional experience, and leadership role were included as covariates. The relationship of all variables involved in the moderation analysis was approximately linear as assessed by visual inspection of the scatterplots after LOESS smoothing. The covariates did not show significant effects on general health in any of the models, indicating that the observed moderating effects remained robust after adjustment for these demographic and occupational factors.

**Fig. 1:**
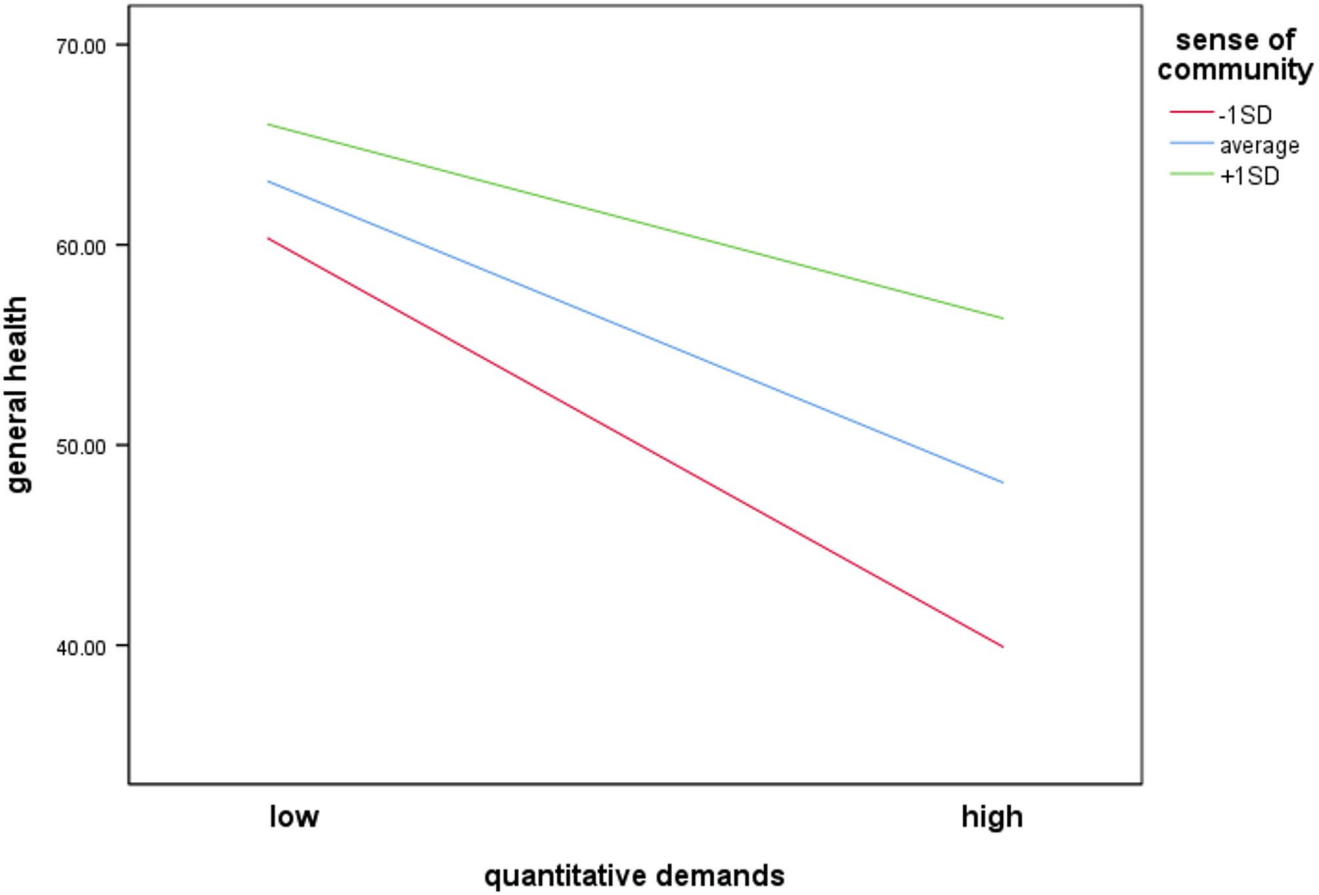
Moderating effect of sense of community on the association between quantitative demands and general health

The overall model of the moderation analysis to determine whether the interaction between quantitative demands and sense of community predicts general health was significant, *F*(8, 283) = 10.94, *p* < .001, predicting 22.58% of the variance. Results show that sense of community moderated the effect between quantitative demands and general health significantly, ΔR² = 1.90%, *F*(1, 283) = 5.34, *p* = .022, 95% CI[0.0013, 0.0122]. The simple slopes indicate that quantitative demands had a negative significant association with general health (*b* = − 0.37, *SE* = 0.07, *p* < .001) and sense of community a positive significant association (*b* = 0.27, *SE* = 0.07, *p* < .001). The interaction term (*b* = 0.01, *SE* = 0.003, *p* = .022) also significantly related with general health, indicating that sense of community moderates the relationship between quantitative demands and general health. Participants who report a stronger sense of community demonstrate a reduced deterioration in general health in face of increasing quantitative demands (Fig. 1).

**Fig. 2:**
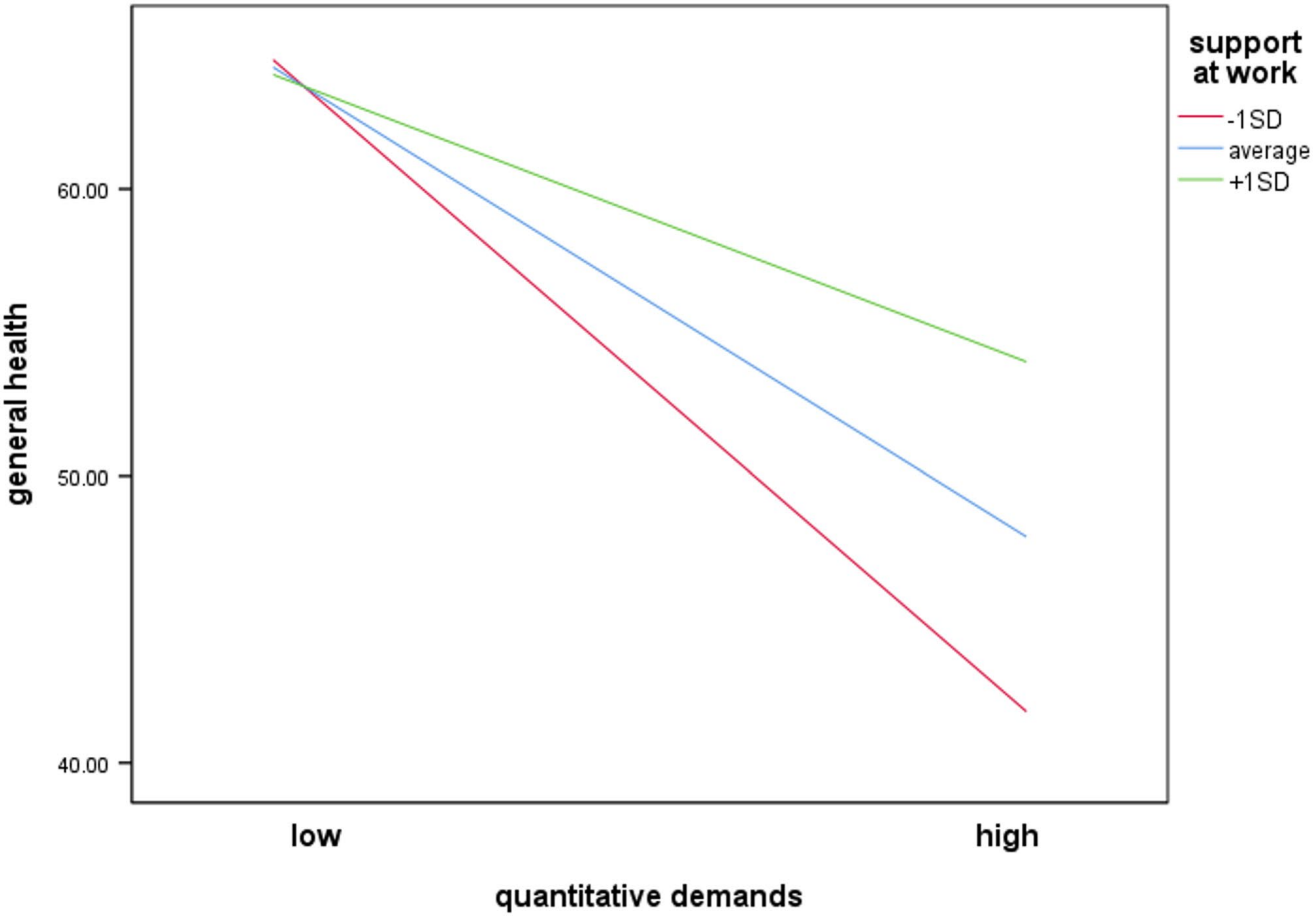
Moderating effect of support at work on the association between quantitative demands and general health

The overall moderation analysis model with support at work as moderator was significant, *F*(8, 284) = 8.26, *p* < .001, predicting 18.79% of the variance. Support at work was a significant moderator of the impact of quantitative demands on general health, ΔR² = 2.06%, *F*(1, 284) = 7.77, *p* = .006, 95% CI[0.002, 0.012]. In this model, there was a negative significant association between quantitative demands and general health (*b* = − 0.41, *SE* = 0.08, *p* < .001) and a positive significant association between support at work and general health (*b* = 0.12, *SE* = 0.06, *p* = .031). The interaction term (*b* = 0.01, *SE* = 0.002, *p* = .006) also significantly related to general health. This indicates that the higher the level of support at work, the lower the deterioration in general health when quantitative demands increase (Fig. 2).

**Fig. 3:**
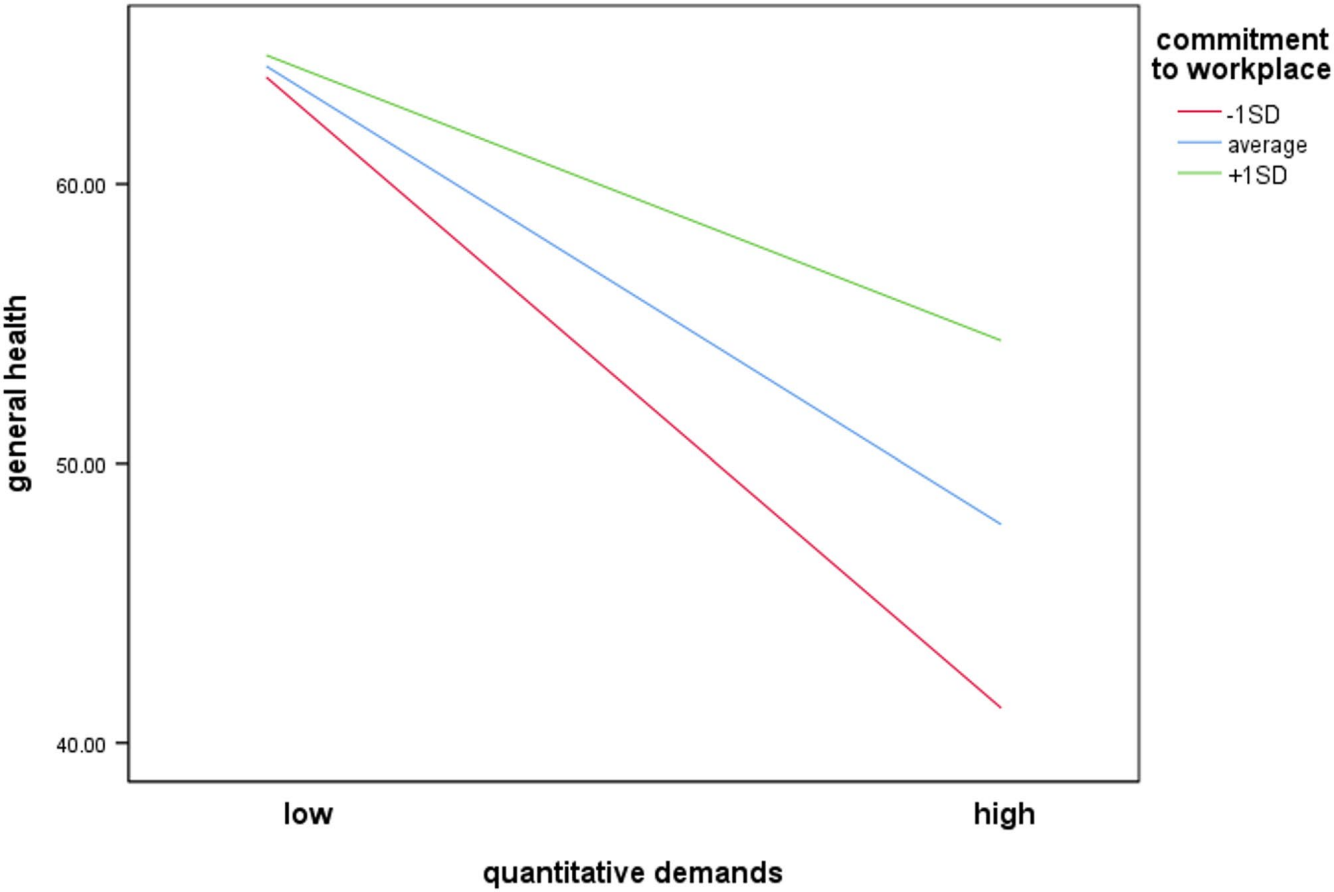
Moderating effect of commitment to workplace on the association between quantitative demands and general health

The overall model of the moderation analysis with commitment to workplace as moderator was significant, *F*(8, 285) = 8.55, *p* < .001. The model explained 18.13% of the variance. Results indicate that commitment to workplace significantly moderated the effect of quantitative demands on general health, ΔR² = 2.00%, *F*(1, 285) = 6.19, *p* = .013, 95% CI[0.001, 0.011]. In this model, there was a negative significant association between quantitative demands and general health (*b* = − 0.41, *SE* = 0.07, *p* < .001) and a positive significant association between commitment to workplace and general health (*b* = 0.13, *SE* = 0.05, *p* = .013). The interaction term (*b* = 0.01, *SE* = 0.002, *p* = .013) also significantly related to general health. This suggests that higher levels of workplace commitment were associated with less deterioration in general health when quantitative demands increase (Fig. 3).

In addition, further models including resilience, self-efficacy, meaning of work, and recognition as moderators were tested, but none of these showed significant interaction effects. The overall models were significant (all R² between 0.16 and 0.17, all ps < 0.001); however, the interaction terms did not explain a significant increase in variance (all ΔR² < 0.01, all ps > 0.10). For resilience, the interaction term with quantitative demands dot not significantly relate to general health (*b* = − 0.08, *SE* = 0.10, *p* = .421). Similar results were observed for self-efficacy (*b* = − 0.08, *SE* = 0.09, *p* = .428), meaning of work (*b* = 0.004, *SE* = 0.004, *p* = .255), and recognition (*b* = 0.002, *SE* = 0.003, *p* = .563). This indicates that these resources did not significantly moderate the association between quantitative demands and general health in the study sample.

## Discussion

With this study it was possible to investigate the impact of job demands on general health of nursing staff in nursing homes in Germany, focusing on the buffering effect of personal and job resources. The results show a significant negative association between quantitative demands and general health among nursing staff. Nurses with higher quantitative demands reported a worse general health, indicating higher strain compared to nurses experiencing lower demands. These findings align with previous research demonstrating that high job demands are a critical source of stress for caregivers [5, 6, 8]. Research indicates that many nursing professionals report experiencing chronic stress. Especially, geriatric nursing staff face particularly high levels of stress, which can result in strain manifested in both physical and psychological symptoms [5]. The results of the present study demonstrate a high workload of nurses in nursing homes. The average score of quantitative demands (66 ± 21) is higher than the reference values of geriatric nurses in Germany (56 ± 19) [44]. Demands at work probably increased due to the additional challenges faced during the COVID-19 pandemic. Staffing shortages, dealing with COVID-19-infected residents and with relatives, and further hygiene measures that needed to be implemented in daily work tasks such as wearing personal protective equipment presented significant challenges for nurses in nursing homes that may have heightened their quantitative workload [15]. Moreover, there were notable gaps regarding support measures to deal with the increased workload [45]. Other studies support the findings of an increased physical and psychological workload during the COVID-19 pandemic [13, 14]. In addition to increased demands during the COVID-19 pandemic, the study indicates that nursing staff experienced high strain reflected in reduced general health during this period. Previous research shows high levels of burnout and mental health problems among nursing staff [46–48]. These findings suggest that the pandemic not only increased job demands but also exacerbated their impact on nurses’ health.

Furthermore, the study demonstrates that high job demands are associated with a deteriorated general health and that certain resources significantly moderate the relationship between job demands and general health. These findings are consistent with the Job Demands-Resources (JD-R) model, which postulates that high job demands can lead to health deterioration, whereas resources can buffer against the negative effects of these demands [28]. The results of the present study indicate that the impact of high workload on general health was reduced with higher job-related resources. A stronger sense of community in the workplace moderated the negative impact of quantitative demands on general health. This suggests that nurses who experience a more supportive and cohesive work environment report better general health even when facing high job demands. Support at work also played a significant buffering role, with higher levels of support mitigating the detrimental effects of high quantitative demands on general health. This highlights the protective role of social resources in stressful work environments. Moreover, the study found that greater commitment to the workplace significantly moderated the negative effect of job demands on general health. Nurses who felt more committed to their workplace experienced less deterioration in general health despite increasing job demands.

The significant moderation effects of these job-related resources can be explained by the assumption that high job demands can lead to negative impacts on general health, but when supported by a strong work community, these demands may feel more manageable. This support could stem from both colleagues and the organizational culture, which emphasizes collaboration. If nurses have a feeling of belonging and that they can rely on support from their colleagues and superiors, it may create a sense of security and the certainty that they can cope with high work demands, reducing the negative impact of workload on general health. Furthermore, if nurses are proud of being part of their organization, job demands could be viewed less negatively and more challenging than threatening; thus, the negative impact of stress could be diminished [49].

The study’s results are aligned with existing literature that underscores the role of job resources in mitigating occupational stress. Previous research has shown that supportive leadership, positive workplace relationships, and job recognition can buffer the adverse effects of stressors such as workload [5, 6]. Our findings extend this understanding to the context of nursing homes, where a higher sense of community, support from colleagues and superiors, and workplace commitment were associated with better health outcomes, even in the face of high workload pressures. This supports the idea that the social and organizational context of the workplace plays a critical role in promoting the well-being of nursing staff. Research suggests that group-level resources such as support at work may not only enhance healthcare employees’ well-being, but also play a particularly important role as a protective factor against high job demands [20].

The lack of significant findings regarding personal resources like resilience and self-efficacy diverges from some previous studies [26, 27]. This could be explained by the fact that the COVID-19 pandemic created unprecedented challenges that might have exceeded the protective capacity of personal resources. The prolonged nature of the pandemic could have led to a depletion of personal resources, reducing their moderating effects [50]. The absence of significant moderation effects for personal resources in this study may also suggest that, in the context of nursing homes, job-related resources might play a more prominent role in health outcomes than personal resources. It could be attributed to the overwhelming nature of job demands in nursing homes. The JD-R model [28] posits that resources must be suitable for the demands at work to buffer against stress. While resilience and self-efficacy are valuable in other contexts, in high-demand workplaces such as nursing homes, job-related resources might be more critical for mitigating health impacts. Personal resources may not be sufficient to buffer against the pervasive structural issues like understaffing and high workload. Peters and colleagues [25] also concluded that job resources seem to be more important than personal resources for nurses’ well-being. Other job-related resources such as meaning of work and recognition did not emerge as significant moderators. This could suggest that while these resources are important for general job satisfaction [6], they may not be as effective in directly mitigating the physical and psychological health impacts of high quantitative demands. In the high-pressure environment of nursing homes, social support and a strong sense of belonging may be more crucial for maintaining general health.

These results imply that the type of resource seems to be relevant regarding the association between resources and strain in the workplace. Future research could investigate how resources vary in different fields of work such as in nursing, in other health professions or in office jobs and which types of resources are most important for general health in different occupations. This study highlights the unique demands of long-term care environments and suggests that systemic changes might be more immediately impactful than interventions focused solely on individual resources such as resilience or self-efficacy. Researchers argue that improving working conditions is essential for sustainable health outcomes, as personal coping strategies alone may not suffice [5]. Interventions targeting the enhancement of social support and sense of community such as interprofessional team building and communication skills training [51], and commitment to the workplace such as appreciative leadership, good working conditions, and opportunities for job crafting [52] could improve health outcomes for nursing staff. Implementing such strategies can not only promote well-being but also improve retention rates. It would be useful to develop more targeted interventions and evaluate their effectiveness to test how well they can improve the health and well-being of nurses. Intervention studies could also provide a direct comparison of measures to improve resources. Although personal resources such as resilience and self-efficacy were not significant in this study, future research could investigate the interactions between personal and organizational resources. The extent to which personal resources could have a stronger buffering effect in certain contexts (e.g. with certain work demands or in combination with organizational resources) could be explored. Moreover, future research could also investigate how the health and wellbeing of nurses influences the quality of care and patient output. This would broaden the practical implications of the study and show how promoting health and well-being among nursing staff could not only improve their own quality of life, but also the quality of care.

## Limitations

The study has several limitations. The cross-sectional design limits the ability to draw causal conclusions, as the relationships between job demands, resources, and general health cannot be definitively established. The moderator analyses show rather low effect sizes; nevertheless, Hayes [36] states that the importance of a moderator should not only be measured by the size of the effect, but also by the theoretical relevance of the moderator and the practical applicability of the results. While conducting separate moderation analyses allows for a clear interpretation of each moderator’s effect, it does not account for potential combined effects among multiple moderators. Moreover, the generalisation of the study results is limited. First of all, the data collection occurred during the COVID-19 pandemic which reflects extreme conditions rather than typical work environments and therefore restricts generalizability to non-crisis situations. Nursing staff faced heightened work demands during the pandemic that exacerbated strain and potentially influenced the accessibility and buffering effects of resources. Consequently, the observed associations between quantitative demands, resources, and general health may reflect the exceptional circumstances of the pandemic, and careful interpretation is required when applied to non-pandemic contexts. Secondly, the study focused on a single federal state of Germany and the response rate of 16.5% may have led to a non-representative sample. The most common reason for nurses not to participate in the study was a high workload. Therefore, the study potentially underrepresents nursing staff who experience the highest levels of stress. Additionally, the self-reported nature of the data may introduce biases such as social desirability or recall bias. Future research could benefit from longitudinal study designs to explore causal pathways and the long-term effects of job demands and resources on general health in this population.

## Conclusions

In conclusion, this study contributes insights into which resources can help nursing staff in long-term care facilities effectively manage their high workload and thus promote their general health. The findings demonstrate the need for nursing homes to develop strategies that enhance job resources in order to buffer the negative effects of high job demands on general health. The identification of the buffering resources sense of community, social support, and workplace commitment provides practical recommendations for improving working conditions and supporting nursing staff in maintaining their well-being. While the focus is often on the detrimental effects of stress, it is essential to recognize that fostering a supportive work environment can enhance nurses’ general health under stress. By implementing strategies to strengthen collaboration and workplace commitment such as team building and appreciative leadership, nursing homes can promote a healthier, more sustainable staff.

## Data Availability

The cross-sectional study data supporting the conclusion of this article is available from the corresponding author upon reasonable request.
